# A Rare Case of Dedifferentiated Retroperitoneal Liposarcoma Presenting As Cardiac-Sounding Chest Pain

**DOI:** 10.7759/cureus.19503

**Published:** 2021-11-12

**Authors:** Bhagat Manku, Parvati Addingadoo, Amjad Ali

**Affiliations:** 1 Medical Education, University Hospital Coventry & Warwickshire, Coventry, GBR; 2 Medicine, University Hospital Coventry & Warwickshire, Coventry, GBR

**Keywords:** retroperitoneal liposarcoma, dedifferentiated liposarcoma, mesenchymal tumors, chemotherapy, mdm2

## Abstract

Retroperitoneal liposarcomas (RPL) are rare mesenchymal tumours with an annual incidence of 2.7 cases per million. Well-differentiated liposarcomas (WDLs) and dedifferentiated liposarcomas (DDLs) are the most common subtype. WDLs are widely known to be low-grade tumours that are less likely to metastasise and easily resected. In contrast, DDLs are high-grade aggressive metastatic tumours with mortality rates between 50% and 70%.

We present an unusual case of a 47-year-old male with a background of hypertension presenting with cardiac-sounding chest pain. Initially managed as an acute coronary syndrome (ACS), he eventually underwent a CT scan which revealed a 20x18x17cm retroperitoneal complex mass with possible infiltrates to the posterior wall of the greater curvature of the stomach. Ultrasound-guided biopsy and subsequent histopathology analysis revealed DDL consistent with MDM2 amplification.

This case highlights how RPL can present with diagnostic difficulties. Multidisciplinary input from haematology, surgery and specialist teams is vital to optimise patient management.

## Introduction

Retroperitoneal liposarcomas (RPL) are rare tumours that contribute to 10%-15% of sarcomas with an annual incidence of 2.7 cases per million [1,2]. They have a peak incidence between the sixth and seventh decade with no specific propensity for age or ethnic group. These tumours have various rates of growth and due to the capacity to grow and expand within the retroperitoneum, their clinical presentation can be late in onset and nonspecific. Such examples include abdominal pain, early satiety, peripheral oedema or some patients may be completely asymptomatic. Musculoskeletal and neurological symptoms may be exhibited secondary to local invasion or compression [3]. Therefore, it is important to elicit a systematic approach within the history and examination to screen for symptoms.

## Case presentation

A previously fit and well 47-year-old lorry driver presented with a three-hour history of central burning chest pain with radiation to the left side of his chest which was associated with one episode of vomiting. His past medical history consisted of hypertension, a body mass index of 40.09, non-alcoholic fatty liver disease and hypercholesterolaemia. He was a non-smoker and drank between three and six units of alcohol per day. There was no relevant family history.

On examination, there was evidence of diaphoresis and raised blood pressure (172/119mmHg); however, all other observations were unremarkable. Despite his ECG showing sinus rhythm with no ischaemic changes, he was started on acute coronary syndrome (ACS) treatment with dual antiplatelets, enoxaparin and glyceryl trinitrate (GTN) spray due to what initially looked like a cardiac clinical presentation; however, the former medications only partially improved symptoms (mainly the GTN spray).

On admission, his blood results were unremarkable and his troponin levels were unconcerning (first level 7ng/L, second level 7ng/L). A thorough review by the Cardiology team ruled out any cardiac cause of his chest pain and the ACS protocol was stopped. Omeprazole was commenced due to the possibility of gastroesophageal reflux as a cause of chest pain, as he was experiencing burning retrosternal discomfort. His chest x-ray was normal with no signs of consolidation or collapse.

Despite normal liver function tests and amylase the patient was still experiencing ongoing right-sided lower chest pain and right upper quadrant pain. Therefore an abdominal ultrasound was requested to rule out any causes for this. This revealed a 15x11cm ill-defined heterogenous lesion that appeared to arise from the upper pole of the left kidney. The liver showed non-specific fatty changes with mild to moderate ascites.

**Figure 1:**
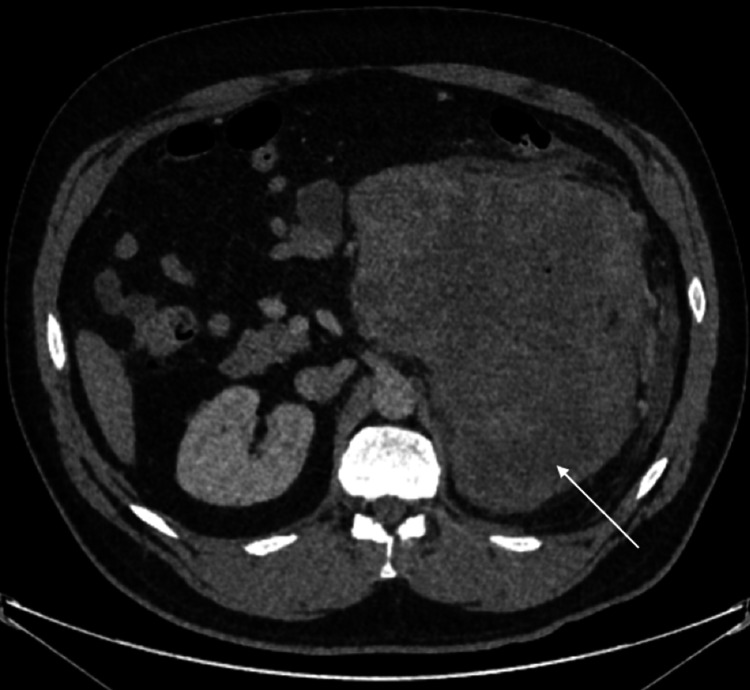
CT scan of the abdomen with contrast demonstrating large retroperitoneal mass encasing the left kidney and vessels (arrow).

A subsequent computerised tomography (CT) scan of his renal tract showed a 24-cm retroperitoneal complex mass with probable infiltrates to the posterior wall of the greater curvature of the stomach. A further staging CT showed significant left-sided retroperitoneal changes encasing the kidney and vessels at the gastrosplenic ligament, extending into the omentum and peritoneal cavity with evidence of haemorrhage within the lesion. There was also a small volume haemoperitoneum within the pelvis, but no metastases (Figure 1).

**Figure 2:**
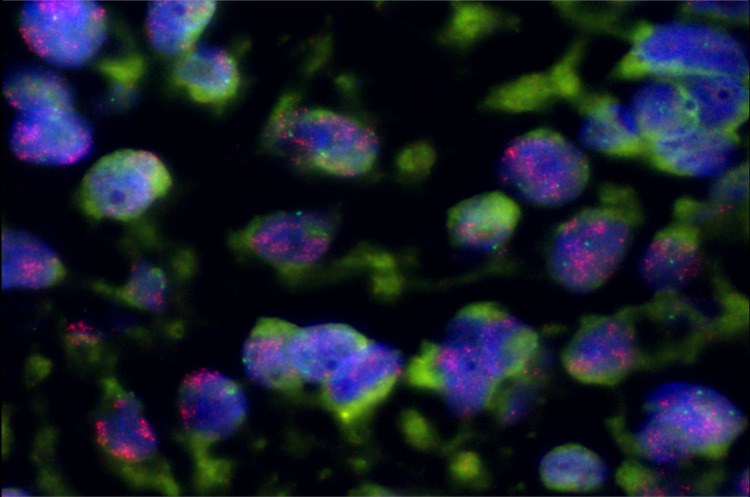
Immunohistochemistry staining of ultrasound-guided core-biopsy of retroperitoneal mass with the proliferation of small round blue cells.

Further investigations were performed to explore the cause of the retroperitoneal mass such as a haematological aetiology. Subsequent ultrasound-guided biopsy revealed dedifferentiated liposarcoma. Immunohistochemistry was positive for tumour markers CD56 and CD138. There was also patchy membranous positivity with CD99 marker (Figure 2).

Due to the size of the tumour at the presentation, it was inoperable. He was commenced on doxorubicin and ifosfamide chemotherapy.

Prior to his second cycle of treatment, he was admitted to the hospital for pyrexia of unknown origin. During this admission he had a further CT scan, which showed that the tumour had increased in size, almost entirely encasing the left kidney as well as the splenic vein and artery. The mass effect caused by this rapid growth displaced the stomach, left adrenal gland, small bowel, coeliac axis and superior mesenteric artery. He is currently being followed up by the sarcoma team and after his third cycle of chemotherapy, imaging will be repeated to reassess for the possibility of surgical resection.

## Discussion

Liposarcomas are classified into four histological groups: well-differentiated, dedifferentiated, myxoid/round cell and pleomorphic liposarcomas as per the World Health Organisation [4]. DDLs are a relatively rare phenomenon where an existing well-differentiated liposarcoma (WDL) gives rise to a high-grade non-lipogenic sarcoma. In this case, the patient was in his 40s; however, DDLs are known to be more prevalent in the sixth decade of life and favour males with the majority arising in the abdominal cavity [5]. Together, WDL and DDL constitute the largest subgroup of liposarcomas [6]. WDL features more mature fibrous adipose tissue and it is known to almost never metastasise. Upon differentiation to DDL, it often displays more aggressive behaviour with higher chances of local recurrence, metastasis and a 50%-70% mortality rate [6,7]. The transformation from WDL to DDL occurs in 20% of first-time retroperitoneal local recurrences and 44% of second-time local recurrences [8]. Unfortunately, poor outcomes are associated with retroperitoneal DDL, particularly those exhibiting myogenic differentiation with rhabdomyoblastic elements [6]. Furthermore, retroperitoneal sarcomas have an overall five-year survival rate between 36% and 58% [9].

A distinguishing feature of WDL and DDL is evidence of a supernumerary ring and giant rod chromosomes that contain amplified oncogenes such as MDM2, CDK4, HMGA2 and TSPAN31 that play a key role in the tumorigenesis of liposarcomas [7,10]. And, 90% of DDL characteristically reveals numerous amplifications in the MDM2 and CDK4 oncogenes, caused by amplification of chromosome 12q [8]. The p53 suppressor is gene is well known to have a vital role in tumour suppression. MDM2 has a direct role in the degradation of the p53 tumour suppressor gene by inactivating the activation domain of p53, therefore, leading to the escape from p53-regulated growth control [11]. MDM2 also behaves as a ubiquitin ligase and facilitates targeted degradation of p53 via proteasomes [7].

Furthermore, poor prognosis has been associated with 1p32 and 6q23 chromosomal amplifications causing increased levels of JUN proto-oncogene and ASK1 which in turn are involved in the dedifferentiation of adipocytes [10]. Other studies have suggested hypermethylated tumours had reduced survival. Histone alterations and gene silencing have also been implicated in the pathogenesis of tumour expansion and dedifferentiation [10].

The approach to a retroperitoneal mass should begin with cross-sectional imaging and there are arguments for both multiple resonance imaging (MRI) and CT. It is generally accepted that the former is more suited for lesions in the extremities and the latter for chest, abdomen, pelvic and retroperitoneal masses. On imaging, DDL is characteristically identified as a retroperitoneal non-lipogenic mass surrounded by abnormal appearing fat. It is generally agreed that upon diagnosis the mass may be surgically removed. Core biopsy is only indicated in tumours that are unlikely to be completely resectable due to vascular involvement [8]

Extremity liposarcomas are commonly treated surgically via limb-sparing procedures; however, it is not as simple for RPLs due the higher rates of recurrence. Surgical resection of retroperitoneal tumours should be considered including resection of any involved adjacent organs; however, co-morbidities, age and asymptomatic DDL may negate the need to intervene surgically [8]. A retrospective study assessed patients with retroperitoneal sarcomas that were surgically managed and found that the rate of recurrence at five years was lower than in historical controls (22% and 41%, respectively); however, surgical morbidity was increased [12].

Systemic approaches to treating DDL have previously been limited to certain chemotherapeutic agents with doxorubicin, gemcitabine and docetaxel being amongst the most popular; however, it is widely accepted that the benefit from chemotherapy for DDL is reported to be minimal [13-15]. Anthracyclines such as doxorubicin are indicated in treating soft tissue sarcomas that are high grade [16]. In combination with ifosfamide, response rates were higher (26.5%) than doxorubicin monotherapy (13.6%) and enhanced progression-free survival (from 4.6 months to 7.4 months) as per the European Organisation for Research and Treatment of Cancer (EORTC) Phase III study. The study did not show any difference in overall survival. Combination therapy has been used with the aim to decrease tumour size to optimise resection in patients fit enough to tolerate its toxicity [16].

Other than the first-line chemotherapy options, there are other options that have been shown to have some benefits. For example, a phase II clinical trial involved treating patients with Palbociclib (a highly selective inhibitor of CDK4 and 6) demonstrated a progression-free survival rate of 66% in patients. However, another study using the same regimen (as well as surgical excision) on a patient with DDL did not have a successful outcome and the patient subsequently developed new lung nodules. Therefore, it is widely accepted that the benefit from chemotherapy for DDL alone is reported to be minimal [17,18].

Recently, advances in molecular biology have led to the development of targeted MDM2 antagonists. One study looked at the use of such an agent (RG7112) in 14 patients with DDL MDM2-amplified liposarcomas, where they received three 28-day cycles of the treatment and found that only one patient had a confirmed partial response, with the remainder showing stable disease [19]. Another study looked at the efficacy of an MDM2 antagonist Selinexor versus the traditional first-line doxorubicin in DDL patient-derived xenografts. Selinexor displayed a greater tumour response compared to doxorubicin (46%-80% and 37%-60% response rates, respectively). Selinexor was also more efficient at displaying apoptotic responses in the xenografts and cell lines compared to doxorubicin [20].

Although surgical resection of WDL and DDL remains the first choice, it remains an unsuitable option for patients with unresectable, unstable and metastatic disease, especially in those with large liposarcomas due to poor outcomes. It is clear there is much development to be made in this area, and until then prognosis remains poor with almost 50% of patients eventually dying with the disease [21]. In the meantime, an MDT approach to diagnosis and management is vital to providing the best outcome possible for such patients with this rare diagnosis.

## Conclusions

RPL have insidious and clinically occult presentations and when they do manifest, can disguise themselves as other common clinical presentations. This in turn can lead to diagnostic difficulty. There are limited cases within literature where retroperitoneal liposarcomas initially present with primarily cardiac-sounding chest pain alone. This highlights an unusual presentation that may not have been previously considered. 

Surgical resection of both WDL and DDL remains the first-line approach in management. Combination chemotherapy regimes with ifosfamide and doxorubicin have shown to improve progression-free survival but have not enhanced overall survival, therefore early detection is crucial. Targeting oncogenes could provide new avenues of treatment. Due to their high recurrence rate, timely follow-up using a multidisciplinary team approach is significantly important in optimising patient outcomes.
